# Editorial Notes

**Published:** 1882-06

**Authors:** 


### EDITORIAL NOTES.

DR. JOHN T. HODGEN, of St. Louis died suddenly and unexpectedly at half past seven o'clock on the evening of April 28th.

THE nestor of American surgeons, Prof. Gross, of Philadelphia, has resigned the chair of surgery in the Jefferson Medical College. As a teacher, writer and surgeon he has had few equals and no superior, and no medical man has been better known abroad or at home. Although of ripe old age he is still healthy, and it is to be hoped that his life may still for many years be spared for the profession of which he has been so bright an ornament.

According to calculations made by the Medical Academy of Paris, there are at present 189,000 doctors scattered over the world. Of these there are 65,000 in the United States; 26,000 in France; 32,000 in Germany and Austria; 35,000 in Great Britain and its colonies; 11,000 in Italy, and 5,000 in Spain. Putting aside pamphlets and memoirs innumerable, it is estimated that 120,000 works have been published on Medical subjects. Of the writers 2,800 are American; 2,600 French; 2,300 German and Austrian, and 2,100 English.

The new code of New York State Medical Society.—At a recent meeting of the Homœopathic Medical Society, of Lancaster, Pa., the following resolution was unamously adopted:

*Resolved*, "That it is the sense of this meeting, that since the practice of homœopathy has established for itself an honorable position in the estimation of the community, against all the opposing forces that the old school could bring to bear against it, there is no advantage or prestige to be derived by homœopathic physicians in consulting with *allopaths*, and therefore the recent action of the *Allopathic* Medical Society of the State of New York in resolving in future to consult with, was entirely gratuitous."

Sir William Ferguson, after a successful operation on a Manchester millionaire, was asked by the patient to name his fee.

"Two hundred guineas."

"Two hundred guineas," exclaimed the patient.

"Yes," said Sir William. "You forget the life-long experience required to give the proper skill, the time and toil of the journey, and the loss of practice in London."

"But you have been only ten minutes about it, said old Dives.

"Oh! if that is your only objection," said Sir William, in his broad Scotch, "the next time I come, I'll keep ye an 'oor under the knife."

Parke, Davis & Co., have issued their Working Bulletin for the specific investigaticn of Quebracho, (aspidosperma quebracho). The physiological action of this new remedy, upon the lower animals, gives paralysis of the respiratory organs and diminished frequency of the heart beat. Upon man in the different forms of dyspnœa, (from emphysema and severe bronchitis, phthisis, periodic asthma and pleuritis), the frequency of breathing has been found to be diminished and the respirations less deep. This action of the drug points to its efficacy in asthma and other diseases of the organs of respiration. We refer our readers to the proper notice under our advertisements.

